# Parental Emotion Socialization and Child Psychological Adjustment among Chinese Urban Families: Mediation through Child Emotion Regulation and Moderation through Dyadic Collaboration

**DOI:** 10.3389/fpsyg.2017.02198

**Published:** 2017-12-18

**Authors:** Zhuyun Jin, Xutong Zhang, Zhuo Rachel Han

**Affiliations:** ^1^Beijing Key Laboratory of Applied Experimental Psychology, National Demonstration Center for Experimental Psychology Education, Faculty of Psychology, Beijing Normal University, Beijing, China; ^2^Department of Human Development and Family Studies, Pennsylvania State University, University Park, PA, United States

**Keywords:** emotion socialization, child emotion regulation, child psychological adjustment, dyadic collaboration

## Abstract

The theoretical model of emotion regulation and many empirical findings have suggested that children’s emotion regulation may mediate the association between parents’ emotion socialization and children’s psychological adjustment. However, limited research has been conducted on moderators of these relations, despite the argument that the associations between parenting practices and children’s psychological adjustment are probabilistic rather than deterministic. This study examined the mediating role of children’s emotion regulation in linking parents’ emotion socialization and children’s psychological adjustment, and whether dyadic collaboration could moderate the proposed mediation model in a sample of Chinese parents and their children in their middle childhood. Participants were 150 Chinese children (87 boys and 63 girls, *M*_age_ = 8.54, *SD* = 1.67) and their parents (*M*_age_ = 39.22, *SD* = 4.07). Parent–child dyadic collaboration was videotaped and coded from an interaction task. Parents reported on their emotion socialization, children’s emotion regulation and psychopathological symptoms. Results indicated that child emotion regulation mediated the links between parental emotion socialization and child’s psychopathological symptoms. Evidence of moderated mediation was also found: supportive emotion socialization and child emotion regulation were positively correlated only at high and medium levels of dyadic collaboration, with child’s psychopathological symptoms as the dependent variables. Our findings suggested that higher-level parent–child collaboration might further potentiate the protective effect of parental supportive emotion socialization practices against child psychopathological symptoms.

## Introduction

Over the past two decades, a burgeoning body of research has focused on the development of children’s psychological adjustment in the context of parental socialization practices (Eisenberg et al., 1998a; Denham et al., 2007). Considerable theoretical and empirical work underlined the central role of emotional competence in child psychological adjustment and suggested that one particular mechanism of the link between parental emotion socialization and child psychological adjustment is through child emotion regulation (Greenberg et al., 1995; Eisenberg et al., 2001; Morris et al., 2007). For example, parents’ supportive socialization may help children obtain skills to regulate negative emotional reactions, thus achieve better adjustment outcomes. By contrast, when children get frustrated, children with parents who respond in a distressed or punitive way may experience prolonged negative emotions or express their frustration in a maladaptive manner (e.g., aggression), which would increase the risk for psychopathological symptoms (e.g., internalizing and externalizing symptoms). Such association between specific socialization practices and child adjustment unfold in day-to-day parent–child interaction, and it has been argued that quality and characteristics of dyadic interaction could influence children’s openness to certain parental emotion socialization practices, and thus influence the effectiveness of such socialization (Darling and Steinberg, 1993). In the current study, we consider parental emotion socialization practices and an important aspect of parent–child dyadic interaction (i.e., dyadic collaboration) concurrently on their associations with child emotion regulation and psychological adjustment among Chinese families with children in their middle childhood.

### The Mediating Mechanism of Child Emotion Regulation

Emotion regulation has been defined as “the extrinsic and intrinsic processes responsible for monitoring, evaluating, and modifying emotional reactions, especially their intensive and temporal features, to accomplish one’s goals” (Thompson, 1994). Many of the models that investigated the association between parental emotion socialization and child’s psychosocial development have examined child emotion regulation as the mediator in the frameworks (e.g., Morris et al., 2007; Han and Shaffer, 2014).

Among the parental emotion socialization practices, the reaction of parents to the negative emotions of their children is an important parenting construct that could directly influence the development of child emotion regulation, because children learn from parents’ responses about which emotions are acceptable and which are not (Eisenberg et al., 1998a). Specifically, supportive parents are aware of children’s negative emotions, they accept them and will help children recognize and manage these negative emotions, for example, by talking to the children about the emotional experience, or working with them in solving the problems inducing negative emotions. In contrast, unsupportive parents view negative emotions as harmful or aversive, they tend to criticize and punish children for expressing negative emotions, in the hope of avoiding or quickly stopping children from expressing negative emotions (Gottman et al., 1996; Lunkenheimer et al., 2007). A substantial amount of evidence documented the relation between parental reactions to children’s negative emotions and child emotion regulation (Cole et al., 2009; Han et al., 2015). For example, researchers indicated that parental supportive emotion socialization (e.g., focusing on children’s emotional experience or the problems that have elicited negative emotions, or encouraging children to express their emotions) could assist children in learning emotion-related knowledge and enhance their emotional awareness, thus facilitate children to develop adaptive emotion regulation skills, whereas parental unsupportive emotion socialization, such as hostility toward and minimization of children’s emotional experience, has been linked to deficiency in child emotion regulation (Eisenberg et al., 1998a).

Further, emotion regulation has been linked to children’s psychological adjustment. For example, many studies showed that better emotion regulation capability was associated with fewer internalizing (e.g., anxiety and depression; McCoy and Raver, 2011) and externalizing symptoms (e.g., aggressive behaviors; Han and Shaffer, 2014) in children. By contrast, children with deficiency in emotion regulation capability may lack the competence to modulate emotional arousal and emotionally driven behaviors and, thus, are at risk of externalizing (e.g., reactive aggression; Eisenberg et al., 2010) and internalizing problems (e.g., depression and anxiety; Eisenberg et al., 1998a). The relations among parental emotion socialization, children’s emotion regulation, and further adjustment are likely to be bidirectional (Eisenberg et al., 1998a; Morris et al., 2007). Specifically, research has showed that while specific parenting practices shape children’s emotion regulation and adjustment, children’s emotional and behavioral problems (e.g., proneness to negative emotions, disruptive behaviors) may in turn induce harsh or insensitive parenting (e.g., Combs-Ronto et al., 2009; Eisenberg et al., 2015). Over time, such mutual effects could build up certain patterns within families where parental emotion socialization would be associated with children’s psychological adjustment through its covariation with children’s emotion regulation capability.

### The Moderating Effect of Parent–Child Dyadic Collaboration

Although previous studies have well established the links between parental emotion socialization behaviors and children’s psychological adjustment via children’s emotion regulation, not all children benefit to the full from parents’ attempts to be supportive. Similarly, some children with parents who display unsupportive emotion socialization have difficulties regulating emotions and may develop psychopathological symptoms, while others do not (Thompson and Meyer, 2007). Such variations might become more salient as children enter middle-childhood and approach adolescence, when they perceive things more independently and may vary more in the extent of openness to parents’ input or have more conflicts with the parents (Kerns et al., 2006). Therefore, the associations among parental emotion socialization, child emotion regulation and child psychological adjustment may not be deterministic, and the characteristics and quality of parent–child dyadic interaction should be considered as contextual factors for these associations (Darling and Steinberg, 1993). While previous research has focused more on specific parenting or parental socialization practices, more attention has been called for on the role of more overarching family functioning or climate (Fosco and Grych, 2013).

Eisenberg et al. (1998b) proposed a theoretical model arguing that the overall quality of parent–child interaction could moderate the links between parental emotion socialization and child emotion regulation. Furthermore, Chen (2017) described based on qualitative research that socialization processes in family context happen as collaborations where children take on parents’ assistance in coping with frustration. Therefore, quality of the collaborative interaction within dyads may shape the association between specific emotion socialization practice and child emotion regulation. Quality of dyadic collaboration, which captures the goal-corrected partnership between a parent and a child, indicates the extent to which they actively contribute their own ideas while seeking joint understanding of each other and acting in reciprocal and mutually supportive ways (Bowlby, 1982; Kerns et al., 2001; Obsuth et al., 2014). Research has indicated that parents’ emotional attitudes toward children in the processes of dyadic interactions influenced children’s openness to specific parenting behaviors, which in turn might change the effectiveness of those parenting practices (Darling and Steinberg, 1993; Kerns et al., 2001). For example, it is possible that the influence of parents’ guidance on facilitating children’s performance (e.g., academic achievement) would be greater among children who feel adequate affection and warmth with parents. However, parents’ cold and disrespectful attitudes may increase children’s resistance to the socialization of their parents, thus attenuate the beneficial effects of even technically correct parenting practices (Darling and Steinberg, 1993).

However, few empirical studies have validated the potential moderating effect of such interaction on the association among parental emotion socialization, child emotion regulation and child’s psychosocial adjustment, with majority of studies only focused on the moderating roles of child characteristics, such as temperament and physiological resilience (e.g., Yagmurlu and Altan, 2010; Perry et al., 2012). Thus, an examination of the interplay between parental emotion socialization and parent–child dyadic collaboration, which illustrates the goal-corrected partnership between parent and child in middle childhood, would provide a more comprehensive understanding of child emotion regulation and the development of psychopathological symptoms (Rutter and Sroufe, 2000; Morris et al., 2007; Han et al., 2015).

### Culture and Family Emotional Process

Although evidence has been accumulating for how parental emotion socialization practices influence children’s emotion regulation and psychological adjustment, most relevant studies were conducted in western cultural contexts. Therefore, many findings may not be readily generalizable to families from other cultural backgrounds because the goals and strategies of emotion regulation and parental socialization could be different according to the cultural norms in specific communities (Cole and Tan, 2006; Friedlmeier et al., 2011). For example, while emotion suppression could lead to negative emotional and social consequences for individuals with mainly Western-European values, its adverse effects were reduced for individuals who embraced more Asian values (Butler et al., 2007).

Such cross-cultural differences may be explained by the fact that the same emotional processes could have different adaptiveness in different cultural contexts (Matsumoto et al., 2008). A classic example is that Eastern cultures usually value connections with others and collective interests (i.e., collectivist), whereas Western cultures have a more independent representation of self (i.e., individualist; Triandis, 1995). Therefore, Eastern cultures may emphasize more the function of emotion in social interactions, and a direct expression of negative emotions is likely to be seen as inappropriate because of the risk of disrupting group harmony (Markus and Kitayama, 1991; Bond, 1993). Empirical evidence has also indicated that such cultural norms could influence how parents socialize their children’s emotional experience (Wang, 2001). Therefore, parental emotion socialization behaviors and their effects on child emotional development should be examined in multiple communities and be understood in specific cultural contexts (e.g., Bornstein, 2012; Suveg et al., 2014).

Moreover, the rapid changes in social and economic contexts in specific societies, such as in China, have been continually shaping parents’ parenting attitudes and practices (Wang and Fivush, 2005; Way et al., 2013). Specifically, the Chinese culture has experienced considerable westernization, and Chinese parents have been having more contact with and understanding about the Western child-centered pattern of child rearing, especially those with well-educated groups (Chang et al., 2003). Compared to traditional Chinese parents who had higher demands on children’s compliance (Bernstein et al., 2005; Xu et al., 2005), contemporary Chinese parents may be more likely to recognize and promote their children’s perspectives and ideas (Sung, 2010). The dyadic collaboration between Chinese parents and children not only reflects the desire for harmony in Chinese families but also embodies the influence of Western culture. Therefore, more studies are needed to understand parental socialization, dyadic collaboration and child development in Chinese families under the dual influences of collectivist cultural traditions and individualist values flowed in during the past decade.

### The Present Study

Collectively, the extant theories and literature suggest that the relationships between parental emotion socialization and child psychological adjustment are likely to vary by the quality of parent–child collaboration. In the present study, we examined a potential mediator and a moderator on the link between parents’ emotion socialization and children’s internalizing and externalizing symptoms in a Chinese middle-childhood sample. In addition, most previous studies only examined children’s emotion regulation via one single instrument (e.g., parental report or observation; Dennis et al., 2010; Suveg et al., 2011), we strived to fill the gaps by using both parental report and the observational task. Behavioral observation allows us to objectively evaluate children’s emotion regulation based upon the same criteria defined by researchers and decrease systematical personal biases (Gardner, 2000). Meanwhile, parental report provides us with a direct way to assess children’s emotion regulation capacities in their everyday lives. We specifically examined the following questions: (1) Are parental emotion socialization associated with Chinese children’s internalizing and externalizing symptoms through children’s emotion regulation? It was expected that parents’ supportive emotion socialization would be positively associated and unsupportive emotion socialization negatively associated with children’s emotion regulation, which in turn would negatively relate to children’s internalizing and externalizing symptoms. (2) Does parent–child dyadic collaboration interact with parents’ emotion socialization in predicting children’s emotion regulation and further, psychological adjustment? It was predicted that parent–child dyadic collaboration would be a moderator of the relation between parents’ emotion socialization and children’s emotion regulation. Specifically, a higher level of parent–child dyadic collaboration was expected to weaken the negative link between parents’ unsupportive emotion socialization and children’s emotion regulation and to strengthen the positive link between supportive emotion socialization and children’s emotion regulation. By contrast, a lower level of parent–child dyadic collaboration was expected to exacerbate the negative effect of parents’ unsupportive emotion socialization on children’s emotion regulation and to weaken the positive link between parents’ supportive emotion socialization and children’s emotion regulation.

## Materials and Methods

### Participants

Data were collected from 150 families as part of a larger project that focused on family emotional context and child emotion regulation. Children (87 boys and 63 girls ranging from 6 to 12 years old, *M*_age_ = 8.54, *SD* = 1.67) participated with one of their parents who self-identified themselves as the primary caregiver (121 biological mothers and 29 biological fathers, *M*_age_ = 39.22, *SD* = 4.07). Most parents were currently married (94.7%), held a college degree (60.7%) or above (33.3%), and were employed with a full-time (74.0%) or part-time job (9.3%). In terms of SES, 76.0% of the families reported living in households that earned equal to or more than the average family income of the city where they were recruited (i.e., approximately $18,500 per year; National Bureau of Statistics of the People’s Republic of China, 2015).

### Procedures

Families were recruited via flyers distributed at local schools and communities or through websites. Interested parents contacted research assistants, and eligible parent–child dyads were invited to the university for a 3-h laboratory visit. Upon arriving at the laboratory, the dyads were introduced to the purpose and procedures of the study and signed informed consent and minor assent forms. They then participated in a series of behavioral and interactive tasks. The current study focused on the observational data from the collaborative task, where the parent and child worked together to draw a picture with an Etch-A-Sketch toy in 4 min. The dyads were given the instruction that parents could only use one knob to draw the horizontal lines, while children could only control the other knob to draw the vertical lines. All behavioral and interactive tasks were videotaped for further analysis. Next, the parent and the child were taken to separate rooms and each filled out a package of questionnaires. All study procedures were approved by the sponsoring university’s Institutional Review Board (IRB).

### Measures

#### Parental Emotion Socialization

Parents filled out the *Coping with Children’s Negative Emotions Scale* (CCNES; Fabes et al., 2002), which evaluates parental emotion socialization practices. The measure presented 12 daily scenarios in which the child experiences negative emotions. For each scenario, six ways of responding to the child’s negative emotions were listed (i.e., distress reactions, punitive reactions, minimizing reactions, expressive encouragement, problem-focused reactions, and emotion-focused reactions), and parents rated how likely it would be for them to respond in each way on a seven-point Likert scale (from 1 “very unlikely” to 7 “very likely”). Scores for the six types of parental response across scenarios were calculated, and two composite scores that summarized parents’ supportive (including problem-focused reactions and emotion-focused reactions) and unsupportive (including distress reactions and punitive reactions) socialization were used in the current study based on the theory that linked particular subscales to reduce the number of analyses (e.g., Suveg et al., 2011; Hurrell et al., 2015). According to previous research, the original version of the CCNES demonstrated satisfactory internal reliabilities (α = 0.69–0.85) and test–retest reliabilities (*r* = 0.56–0.83; Eisenberg et al., 1999; Fabes et al., 2002). The Chinese version of the CCNES also demonstrated acceptable internal reliabilities regarding all subscales (α = 0.68–0.75) and the supportive and unsupportive indexes (α = 0.85–0.91) (Tao et al., 2010; Han et al., 2015). The internal reliabilities were good for both supportive (α = 0.90) and unsupportive (α = 0.86) indexes in the current study.

#### Parent-Report Child Emotion Regulation

Parents reported their children’s emotion regulation capacity via the *Emotion Regulation Checklist* (ERC; Shields and Cicchetti, 1997). The measure consists of 24 items and yields two subscales: (1) Emotion Regulation, which measures parents’ perception of children’s appropriate emotional expression, empathy, and emotional self-awareness (e.g., “Responds positively to neutral or friendly approaches by peers”) and (2) Liability/Negativity, which assesses parents’ perception of children’s emotional liability, inflexibility and dysregulated negative affect in daily life (e.g., “Exhibits wide mood swings). Parents rated the extent to which the descriptions accurately reflected their children’s emotions or behaviors on a four-point Likert scale (from 1 “not at all like this” to 4 “exactly like this”). Given that our developed theoretical framework focused more on the processes central to children’s adaptive control and regulation of emotions rather than children’s negative emotionality, and that CCNES may be more directly relate to the ways children coping with their emotions arousals (e.g., adaptive expression, suppression) rather than directly influence children’s negative emotionality (Cole et al., 2009; Han et al., 2015), the emotion regulation subscale was used. Satisfactory internal reliabilities were found in previous studies for the original version (α = 0.83–0.96; Shields and Cicchetti, 1997) as well as the Chinese version (α = 0.69–0.94; Chang et al., 2003; Han et al., 2015) of ERC. In the current study, Cronbach’s α was 0.64 for the emotion regulation subscale.

#### Observed Child Emotion Regulation

The Child Emotion Regulation Scale measured the extent to which children adaptively regulated and controlled their emotions and reactions. It was coded on a seven-point Likert scale according to the videotapes of the 4-min collaborative drawing task. Indicators of adaptive emotion regulation included that children were able to recover quickly from intense positive or negative emotions, and that children expresses appropriate emotions according to the situation, etc. Indicators of poor emotion regulation included that children exhibited wide mood swings or had difficulty managing emotional intensity, etc. Children who scored low (e.g., 1) on this scale exhibited almost no or not any instance of regulated emotion. Children who scored high (e.g., 7) on this scale exhibited well adaptive emotions and reactions in the task. The coding systems were translated and adapted to the current study from previously developed observational codes (Sroufe et al., 2005). We randomly selected 10 percent of the video recordings and double coded them to calculate the intra-class correlation coefficient of the scale, and the inter-coder reliability coefficients was 0.94.

#### Child Internalizing and Externalizing Problems

Children’s internalizing and externalizing problems were assessed through the Child Behavior Checklist (CBCL; Achenbach, 1991). The CBCL is a parent-report measure of children’s psychological well-being that consists of 118 items. Parents rated the frequency of their children’s behaviors or symptoms for each item on a three-point Likert Scale (from 0 “not true” to 2 “very true or often true”). Scores for internalizing (including somatic complaints, withdrawn behaviors, and anxious/depressed symptoms) and externalizing (including delinquent behaviors and aggressive behaviors) problems were derived from a series of more specific subscales regarding children’s psychopathological symptoms. Extensive evidence has supported the psychometric properties of the original CBCL, and its norms have been established based on data collected from various cultures (e.g., Achenbach, 1991; Crijnen et al., 1999). The Chinese version of the CBCL has been widely used and has demonstrated good reliability and validity (Chen et al., 1999; Leung et al., 2006). In the current study, Cronbach’s α was 0.87 for internalizing problems and 0.88 for externalizing problems.

#### Dyadic Collaboration

Parent–child dyadic collaboration in the present study was assessed through behavioral observation by a group of trained research assistants of videotapes of the 4-min collaborative drawing task. As previous studies have indicated that macrocoding may be better suited for studying family processes related to interactive dynamics and relational concepts (Kerig and Lindahl, 2000), we adopted macrocoding in order to synthesize the entire process of collaborative interaction and code parent–child interaction behaviors of interest in a larger context. Overall, this scale measured the extent to which the parent–child dyad worked together effectively and constructively, and it captured: whether the dyad was seeking joint understanding of each other and the eagerness and receptiveness of their cooperation. Indicators of low collaboration included that the dyads showed consistent refusal or inability to cooperate during the task, either in the way of opposition (e.g., actively undermining the collaboration), or in the way of distancing (e.g., active avoidance of communication). By contrast, indicators of high collaboration on this scale included exhibiting eagerness to cooperate and strived for mutual understanding, such as responding cooperatively to suggestions, actively and patiently explaining their points of view and genuinely asking the other to clarify their ideas. Parent–child dyadic collaboration was coded on a seven-point Likert scale ranging from -3 to 3, with -3 representing a very low quality of collaboration and 3 representing a very high quality of collaboration. Research assistants first rated the level of dyadic collaboration independently and then conferenced the final score for each family. The intra-class correlation coefficient of the scale was calculated with all coders’ scores on a randomly selected 10% of the whole sample. Within the randomly selected coding sample, the inter-rater reliability was 0.94.

### Analytic Plan

**FIGURE 1:**
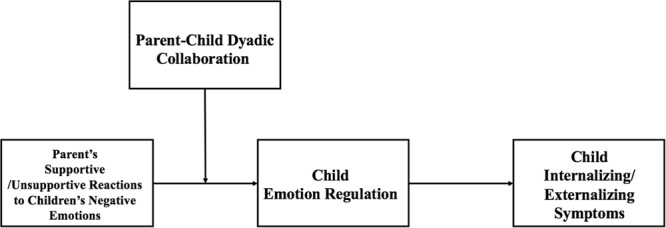
The moderated mediation model.

First, the preliminary analyses examined the descriptive statistics and correlations among study variables and demographic characteristics. Next, mediation models and moderated mediation models were tested using the SPSS PROCESS macro with bootstrapping methods, following procedures recommended by Hayes (2013). Five thousand bootstrap resamples were used to generate 95% confidence intervals for estimating the size and significance of the effects. All studied variables were centered before testing mediational and moderated mediation models. Based on theoretical considerations, we first examined whether parent report and observed child emotion regulation mediated the associations between parents’ supportive/unsupportive reactions and children’s internalizing/externalizing symptoms; then, parent–child dyadic collaboration was tested as a moderator of the association between parental reactions and child emotion regulation in the mediations models (**Figure 1**).

## Results

### Preliminary Analyses and Descriptive Statistics

Rates of missing data ranged from 0 to 2.7%, and all missing data were due to participant non-response (e.g., deliberately or accidentally not responding to certain items). **Table 1** shows the mean scores, standard deviations, and correlations among study variables. Parents’ supportive reactions to children’s negative emotions were negatively correlated with children’s internalizing and externalizing symptoms, whereas parents’ unsupportive reactions were negatively correlated with children’s internalizing symptoms. Parents’ supportive reactions were positively and unsupportive reactions were negatively associated with parent-report children’s emotion regulation. Parent-report children’s emotion regulation was negatively correlated with children’s internalizing and externalizing symptoms. No significant correlation was observed between observed children’s emotion regulation and other study variables. No significant correlation was observed between parent–child dyadic collaboration and other study variables.

**Table 1:** Means, standard deviations, and bivariate correlations of study variables.

Variable	*M*	*SD*	1	2	3	4	5	6	7
(1) Supportive reactions	11.66	1.31							
(2) Unsupportive reactions	5.73	1.54	–0.28^∗∗^						
(3) Parent-report child ER	27.15	2.74	0.38^∗∗^	–0.21^∗^					
(4) Observed child ER	5.97	0.96	0.10	–0.03	–0.09				
(5) Internalizing symptoms	54.49	9.71	–0.24^∗∗^	0.22^∗∗^	–0.39^∗∗^	0.04			
(6) Externalizing symptoms	53.68	9.02	–0.18^∗^	0.13	–0.39^∗∗^	–0.08	0.58		
(7) Dyadic collaboration	1.26	1.14	0.06	–0.12	–0.04	0.36	0.05	–0.01	
(8) Child age	5.84	1.67	–0.10	–0.06	0.04	0.14	–0.11	–0.27^∗∗^	0.08

*ER, emotion regulation; ^∗^*p* < 0.05; ^∗∗^*p* < 0.01.*

The preliminary analyses examined whether the study variables differed by parents’ gender, children’s age, gender, and family social-economic status (SES). The results showed that child age was negatively associated with children’s externalizing symptoms (*r* = 0.27, *p* < 0.01). Additionally, parents of girls reported using more supportive reactions to children’s negative emotions [*t*(148) = 2.11, *p* < 0.05]. Parents’ gender and family SES, indexed by annual household income, was not significantly associated with any study variable. Therefore, children’s age and gender were controlled for subsequent analyses.

### Mediation Analyses

Tests of mediation models showed that parents’ supportive reactions to children’s negative emotions were indirectly related to children’s internalizing symptoms via parent-report children’s emotion regulation, such that higher levels of supportive reactions were associated with better emotion regulation, which in turn was associated with fewer internalizing symptoms (indirect effects point estimate = -0.14, *SE* = 0.04, 95% CI = -0.23 to -0.06). The same mechanism also stood for the association between parents’ supportive reactions and children’s externalizing symptoms (indirect effects point estimate = -0.14, *SE* = 0.04, 95% CI = -0.24 to -0.06). Similarly, parents’ unsupportive reactions to children’s negative emotions were indirectly associated with children’s internalizing symptoms via parent-report children’s emotion regulation, such that higher levels of unsupportive reactions were associated with children’s poor emotion regulation, which in turn was associated with more internalizing symptoms (indirect effects point estimate = 0.07, *SE* = 0.03, 95% CI = 0.02 to 0.15). The same mechanism also stood for the association between parents’ unsupportive reactions and children’s externalizing symptoms (indirect effects point estimate = 0.07, *SE* = 0.04, 95% CI = 0.02 to 0.17). However, the indirect effect was not significant for observed children’s emotion regulation as mediators. **Table 2** details the standardized estimates, errors and confidence intervals for the significant mediation models.

**Table 2:** Standardized estimates, errors, and confidence intervals for significant mediational analyses.

	*a* path (β, *SE*)	*b* path (β, *SE*)	*c* path (β, *SE*)	*c′* path (β, *SE*)	95% CI of the indirect effect
Supportive reactions- Child ER-Internalizing symptoms	(0.39, 0.08)	(–0.34, 0.08)	(–0.24, 0.08)	(–0.15, 0.08)	–0.23 to -0.06
Supportive reactions-Child ER-Externalizing symptoms	(0.39, 0.08)	(–0.35, 0.08)	(–0.18, 0.08)	(–0.10, 0.08)	–0.24 to -0.06
Unsupportive reactions-Child ER-Internalizing symptoms	(–0.20, 0.08)	(–0.36, 0.08)	(0.22, 0.51)	(0.14, 0.08)	0.02 to 0.15
Unsupportive reactions-Child ER-Externalizing symptoms	(–0.20, 0.08)	(–0.38, 0.07)	(0.13, 0.08)	(0.04, 0.07)	0.02 to 0.17

*Child ER, parent report child emotion regulation.*

### Moderated Mediation Analyses

Moderated meditational tests were conducted on previously significant mediational models (Hayes, 2013). Moderated meditation models were tested to examine whether parent–child dyadic collaboration moderated the mediation models, specifically the links between parents’ supportive or unsupportive reactions and parent-report children’s emotion regulation. Preacher et al. (2007) noted that a moderating effect is demonstrated by the significant interaction of the independent variable and the moderator with the bootstrapped confidence intervals not containing zero.

For the models with parents’ supportive reactions as the independent variable and children’s internalizing or externalizing symptoms as the dependent variable, we found significant moderating effects of parent–child dyadic collaboration on the link between parents’ supportive reactions and parent-report children’s emotion regulation (i.e., the mediator; for the model with internalizing symptoms, β = 0.21, *t* = 2.51, *p* < 0.05; for externalizing symptoms, β = 0.21, *t* = 2.51, *p* < 0.05). **Table 3** presents the bootstrapping estimates and slope coefficients for the conditional indirect effects of parental reactions on child psychopathological symptoms. For the model with either child internalizing or externalizing symptoms as dependent variables, parents’ supportive reactions were positively related to parent-report children’s emotion regulation only at medium and high levels of parent–child dyadic collaboration.

**Table 3:** Bootstrap estimates of indirect effects at -1 *SD*, mean, and +1 *SD* levels of the moderator.

*SD* level	Indirect effect (β, Boot *SE*)	95% CI
**Supportive reactions-Child ER-Internalizing symptoms**		
–1 *SD*	–0.05 (0.04)	–0.15 to 0.02
Mean	–0.12^∗∗^ (0.04)	–0.21 to -0.06
+1 *SD*	–0.20^∗∗∗^ (0.06)	–0.33 to -0.10
**Supportive reactions-Child ER-Externalizing symptoms**		
–1 *SD*	–0.05 (0.05)	–0.16 to 0.02
Mean	–0.13^∗∗∗^ (0.04)	–0.22 to -0.06
+1 *SD*	–0.20^∗∗∗^ (0.06)	–0.34 to -0.10

*Child ER, parent report child emotion regulation; ^∗^*p* < 0.05; ^∗∗^*p* < 0.01; ^∗∗∗^*p* < 0.001.*

For the models with parents’ unsupportive reactions as the independent variable and children’s internalizing or externalizing symptoms as the dependent variable, no significant moderating effect of parent–child dyadic collaboration was found for the link between unsupportive reactions and parent-report children’s emotion regulation (for the model with internalizing symptoms, β = -0.08, *t* = -0.93, *p* = 0.35; for the model with externalizing symptoms, β = -0.08, *t* = -0.93, *p* = 0.35).

## Discussion

A growing body of Western research has supported the link between parents’ emotion socialization behaviors and child psychopathological symptoms (Eisenberg et al., 1998a; Silk et al., 2011), as well as children’s emotion regulation as a possible underlying mechanism of the link (Morris et al., 2007; Han and Shaffer, 2014). Meanwhile, theories and related empirical evidence have also indicated the possible variability of the above associations in different families (Eisenberg et al., 1998b; Han et al., 2015). Compared with the Western culture which encourages children to express their feelings and individual independence, Chinese tradition emphasizes the inhibition of emotions and children’s obedience to parents (Butler et al., 2007; Chao, 2011). Whereas, as the larger context of Chinese society has been of westernization during the past decade (Xu et al., 2005), Chinese parents and children have been influenced by both collectivism and individualism, it is worth investigating that whether the relevant Western theories and findings on the relations between family emotional processes and children’s adjustment can be generalized to contemporary Chinese context.

This study aimed to explore whether child emotion regulation served as the mediator of the links between parental supportive/unsupportive emotion socialization and child psychopathological symptoms and further investigated whether these links were moderated by parent–child dyadic collaboration in a sample of Chinese parents and their children in their middle childhood. The results revealed that parent-report children’s emotion regulation mediated the association between parental supportive and unsupportive reactions to children’s negative emotions and children’s internalizing and externalizing symptoms. Moreover, the levels of dyadic collaboration between parents and children moderated the link between parental supportive reactions and parent-report children’s emotion regulation in the moderated mediation models. Although these findings could not inform us on causal relationships over time, they provided a cross-sectional picture of the associations between family processes and children’s emotion regulation and adjustment, which could be established patterns within families over years of mutual influences between parent and child.

First, our results relating to the mediational models supported the tripartite model proposed by Morris et al. (2007) that indicated that parental emotion socialization practices may be related to parent-report children’s regulation of the intensity and expression of emotions and, thus, associated with children’s psychological adjustment, including internalizing and externalizing symptoms. Our findings were consistent with the studies that focused on Western families (e.g., Eisenberg et al., 2001). Although the current study was not a comparative study across cultures, it might be that in contemporary Chinese urban population, especially in well-educated families, the influence of parental emotion socialization on children’s development may not be drastically different from that of the West. In other words, our findings supported that the family emotional process in this similar Chinese samples might be similar to the patterns in western families (Chang et al., 2003; Tao et al., 2010). Specifically, our findings were consistent with previous studies that documented the links between parental supportive/unsupportive reactions to children’s negative emotions and children’s adjustment (e.g., Tao et al., 2010), the link between parental reactions and children’s emotion regulation, as well as the link between children’s emotion regulation and psychological adjustment among Chinese samples (e.g., Han et al., 2015; Li and Han, 2016). Similar to what has been found in western samples (Eisenberg et al., 1998a; Cole et al., 2009), Chinese parents’ comforting and problem-solving oriented practices seem to provide external support for children’s regulation of negative emotions and facilitate children’s use of adaptive self-regulation strategies, such as distracting themselves from the current negative experience. By contrast, when Chinese parents react unsupportively to children’s negative emotions and threaten children with punishment, they may fail to promote children’s acceptance of the emotion-arousal situations or equip children with effective emotion regulation strategies. In turn, when children’s emotion regulation capability is limited, their experience of negative emotions could become more intense and dysregulated, escalating the maladaptive patterns in the family (Eisenberg et al., 1998a; Thompson and Meyer, 2007).

However, the results showed that observed child emotion regulation did not mediate the relations between parental reactions to children negative emotions and children’s internalizing/externalizing symptoms. Although behavioral observation is an important way of measurement, it may only catch children’s regulatory behaviors in a specific laboratory context, while parental report may be able to provide a more comprehensive picture of children’s behaviors in daily life. Our findings supported previous theoretical consideration that behavior observations do not necessarily yield similar findings to those derived from the more natural measurement such as parental report (Gardner, 2000). It is also possible that compared with the observational tasks adopted in other studies (e.g., disappointment task, frustrating task; Dennis et al., 2010; Morris et al., 2011), the collaborative task employed in our study was not very emotion-inducing, thus did not provide enough opportunity for observing children’s emotion regulation. In comparison, parents might be able to inform us about children’s emotional response to various daily life events (e.g., losing a favorite toy, having conflicts with peers) and their regulatory competence.

The findings from the moderated mediation analyses partially supported our second hypothesis, showing that the link between parents’ supportive reactions and parent-report child emotion regulation was stronger when the parent and child showed higher levels of mutual understanding and constructive collaboration but was weakened in the context of lower levels of dyadic collaboration. The findings provided one of the first empirical evidence to support the perspective that the quality of a parent–child interaction can moderate the links between parental socialization and child outcomes (Darling and Steinberg, 1993; Kerns et al., 2001) and contributed to the current literature by facilitating a more integrated understanding of how parent–child dyadic collaborations could influence child emotional development and psychosocial adjustment.

Previous studies have found that children who had more smooth dyadic collaboration or a more optimal interaction with parents demonstrated more positive emotional and behavioral outcomes and showed greater openness to specific parenting practices (Kerns et al., 2001; Demby et al., 2017). In addition, a previous study with Chinese samples indicated that various family processes are interconnected and need to function well to promote children’s development (Han et al., 2015). Our results provided further specific findings that good parent–child collaboration played a key role in amplifying the positive effect of Chinese parents’ supportive emotion socialization practices on child emotion regulation development.

Traditionally in Chinese families, parents and children could be rather emotionally distant due to the status hierarchy (Xu et al., 2005). In such families, children’s obedience and compliance was viewed as basic respect to their parents, and the parents would pass on norms and experiences as experienced instructors (Bernstein et al., 2005; Xu et al., 2005). However, due to the growing acculturation and globalization, Chinese parents are increasingly open to the Western child-centered pattern of child rearing and Chinese children are also increasingly looking forward to be treated as equal with their parents (Kagitcibasi, 2005; Zhang, 2007). It is likely that the juxtaposition of individualism and collectivism has affected the interactive patterns in Chinese families, and the current optimal dyadic collaboration between parents and children may value both their joint understanding with each other as well as their respectful negotiation aimed at a meeting of minds. It is possible that a collaborative parent–child interaction, as the index of the attainment of goal-corrected partnership within the dyad, would enhance children’s benefits from supportive parenting behaviors (Moss et al., 2009). Instead of physical proximity, parents’ availability becomes the set goal of such partnership in middle childhood (Bowlby, 1982; Ainsworth, 1990). Such availability helps a child establish a sense of confidence that parents are open to communications and responsive in case of need, which would improve parents’ effectiveness to socialize their children (Kerns et al., 2001). In Chinese families with higher levels of dyadic collaboration, parents and children are more likely to confidently state their opinions, genuinely ask each other to share the reasoning behind their perspective, and at the same time, may have a warmer manner and a higher mutual understanding of each other’s verbal and non-verbal expression. Through these positive dimensions, Chinese children can infer the positive emotional attitudes of their parents, which could lead to their greater openness to parents’ supportive emotion socialization practices (Darling and Steinberg, 1993). Conversely, a less optimal parent–child collaboration might increase Chinese children’s resistance to parents’ supportive socialization, which would weaken the beneficial effects of these positive practices (Darling and Steinberg, 1993).

Interestingly, in contrast to the models for parental supportive reactions, parent–child dyadic collaboration did not moderate the link between parental unsupportive reactions and child emotion regulation. It appeared that, in our sample, the relation between unsupportive parenting practices and child emotion regulation did not vary regarding the level of dyadic collaboration. When Chinese children were exposed to parents’ unsupportive emotion socialization, the negative effect might have been difficult to buffer by a collaborative interaction pattern within the dyads.

In the current sample, Chinese parents’ reactions to the negative emotions of their children, such as invalidation and punishment, were negatively associated with children’s emotion regulation and adjustment in middle childhood. For children at this age, although they may have been equipped with greater abilities of self-regulation and may have had access to more sources of extrinsic assistance and support for emotion regulation (e.g., from teachers, peers, etc.), they still mainly depend on parents’ related reactions and behaviors to internalize the appropriateness of their feelings and expressions (Zeman et al., 2006; Thompson and Meyer, 2007). When Chinese parents react punitively and negatively to their emotions, children learn from theses reactions that their feelings are wrong and invalid and tend to suppress negative emotions in the moment to evade punishment. These unexpressed or “stored” negative emotions may result in more intense expressions when similar circumstances arise and become more difficult to handle. Over time, these children may develop maladaptive strategies for managing emotions and emotionally driven behaviors in evocative social situations, thus at high risk for psychological maladjustment (Fabes et al., 2002; Tao et al., 2010). Although such association was not buffered by dyadic collaboration, future research would be needed on what other mechanisms may help children build resilience when exposed to unsupportive socialization practices from parents. In addition, it is important to separately study the construct of supportive and unsupportive parenting behaviors and to consider the differences in the variability of the impact of parental supportive and unsupportive behaviors on children in middle childhood.

Although these findings provided important evidence for our understanding of the variability of the associations between parental emotion socialization behaviors and child psychopathology, several limitations need to be acknowledged. First, the present study looked into the associations of interest within a cross-sectional design, which prevented any inference about the causal or bidirectional relations among the study variables. Therefore, the results should be interpreted with caution, and future longitudinal studies are needed to further elucidate the role of various family processes in the development of child emotion regulation and psychosocial adjustment, and whether those constructs shape each other bi-directionally over time. Another major limitation is the measurement of our key variables. Although parent–child dyadic collaboration was obtained via behavioral coding, parental emotion socialization behaviors, child emotion regulation and child psychopathological symptoms were measured only through parental report, which may lead to a potentially overestimated relation between these variables. And the constructs we studied were mostly detected through single measuring instruments. Future work would be more informative if researchers incorporated multiple instruments and informants (e.g., spouse’s report of parenting behaviors, teacher report of child adjustment) for these variables. Additionally, the present study sample was composed of urban, highly educated, Chinese families; further replications with groups of a lower educational level (e.g., families of peasant migrants) are required to ensure the generalizability of the findings. Last, in this study, we mainly focused on the moderating effect of parent–child dyadic collaboration; thus, it will be necessary to conduct more comprehensive investigations of how other family interpersonal dynamics, such as inter-parental conflicts, could play a role in child emotional development and psychosocial adjustment, which would bridge the existing gaps in the literature (Davies and Woitach, 2008).

## Conclusion

Despite these limitations, our findings revealed important information on the pathway through which parental emotion socialization influenced children’s psychological wellbeing. We suggest that more research and practical work should focus on children’s emotion regulation development, because it could be the mechanism through which parenting practices shape children’s psychosocial development. The current study also informed prevention and intervention to build up adaptive emotion regulation skills among children exposed to negative parental emotion socialization. In addition, our findings justified the important role of dyadic collaboration in moderating the specific pathways that link parental emotion socialization and child emotion regulation. We encourage parents to not only provide supportive reactions, such as problem-focused and emotion-focused reactions, when children express negative emotions, but also to have an active empathic and collaborative interaction with children in order to amplify the positive effect of supportive parenting practices. Furthermore, the present study indicates that the relations among parental emotion socialization, child emotion regulation and child adjustment are non-deterministic. Therefore, it is necessary to consider the influence of various family processes as well as the social context on these associations.

## Ethics Statement

This study was carried out in accordance with the recommendations of ‘Institutional Review Board (IRB) of School of Psychology, Beijing Normal University, China’ with written informed consent from all subjects. All subjects gave written informed consent in accordance with the Declaration of Helsinki. The protocol was approved by the ‘Institutional Review Board (IRB) of School of Psychology, Beijing Normal University, China.’

## Author Contributions

Every author has made an important contribution in writing this paper, ZJ took the responsibiliy as first author and led the writing work.

## Conflict of Interest Statement

The authors declare that the research was conducted in the absence of any commercial or financial relationships that could be construed as a potential conflict of interest. The reviewer AA and handling Editor declared their shared affiliation.
